# Reduced *CYFIP1* in Human Neural Progenitors Results in Dysregulation of Schizophrenia and Epilepsy Gene Networks

**DOI:** 10.1371/journal.pone.0148039

**Published:** 2016-01-29

**Authors:** Rebecca A. Nebel, Dejian Zhao, Erika Pedrosa, Jill Kirschen, Herbert M. Lachman, Deyou Zheng, Brett S. Abrahams

**Affiliations:** 1 Department of Genetics, Albert Einstein College of Medicine, Bronx, New York, United States of America; 2 Department of Neurology, Albert Einstein College of Medicine, Bronx, New York, United States of America; 3 Department of Psychiatry and Behavioral Sciences, Albert Einstein College of Medicine, Bronx, New York, United States of America; 4 Department of Medicine, Albert Einstein College of Medicine, Bronx, New York, United States of America; 5 Department of Neuroscience, Albert Einstein College of Medicine, Bronx, New York, United States of America; UTHSCSH, UNITED STATES

## Abstract

Deletions encompassing the BP1-2 region at 15q11.2 increase schizophrenia and epilepsy risk, but only some carriers have either disorder. To investigate the role of *CYFIP1*, a gene within the region, we performed knockdown experiments in human neural progenitors derived from donors with 2 copies of each gene at the BP1-2 locus. RNA-seq and cellular assays determined that knockdown of *CYFIP1* compromised cytoskeletal remodeling. FMRP targets and postsynaptic density genes, each implicated in schizophrenia, were significantly overrepresented among differentially expressed genes (DEGs). Schizophrenia and/or epilepsy genes, but not those associated with randomly selected disorders, were likewise significantly overrepresented. Mirroring the variable expressivity seen in deletion carriers, marked between-line differences were observed for dysregulation of disease genes. Finally, a subset of DEGs showed a striking similarity to known epilepsy genes and represents novel disease candidates. Results support a role for *CYFIP1* in disease and demonstrate that disease-related biological signatures are apparent prior to neuronal differentiation.

## Introduction

The identification of novel disease loci through genome-wide characterization of copy number variations (CNVs) has been important in many regards, including the discovery of genotype-phenotype relationships and genetic counseling. Personalized treatment, however, requires a clear understanding of underlying mechanisms, including which genes within disease-associated loci contribute to risk and how effects are mediated across development. Given that penetrance is often incomplete and expressivity is highly variable with CNVs, the ability to model this complexity is likewise critically important.

In this context, the breakpoint 1 to breakpoint 2 deletion region at 15q11.2 (BP1-2), associated with increased risk for schizophrenia and epilepsy, is exemplary [1–3]. The deletion spans ~ 500 kb and encompasses four genes: tubulin, gamma complex associated protein 5 *(TUBGCP5*), cytoplasmic FMR1 interacting protein 1 (*CYFIP1*), non imprinted in Prader-Willi/Angelman syndrome 2 (*NIPA2*), and non imprinted in Prader-Willi/Angelman syndrome 1 (*NIPA1*). The deletion is common in the general population, occurring at a frequency of ~ 1/500, and is often transmitted to affected children by clinically unaffected parents [4]. While a growing body of work suggests that altered *CYFIP1* expression impacts neuronal morphology and function [5–11], relatively little has been done to evaluate the possible impact of reduced expression at earlier developmental time points, particularly in a human model system. One study using induced pluripotent stem cells (iPSCs) from BP1-2 deletion carriers determined that reduced *CYFIP1* dosage resulted in cell polarity defects in neural rosettes [12]. However, genome-wide transcriptional profiling was not performed in this study, and inter-individual differences, a defining feature of BP1-2 deletions, were not evaluated.

To investigate an early developmental role for *CYFIP1* in disease and explore inter-individual variation in a human system, we developed a knockdown model using lentiviral shRNAs in neural progenitor cells (NPCs) derived from human iPSCs with two copies of each gene at the BP1-2 interval (BP1-2 copy number neutral). NPCs are proliferating cells that resemble radial glia and have the capacity to differentiate into neurons, astrocytes, and oligodendrocytes. In support of their use, work comparing NPCs derived from individuals with idiopathic schizophrenia and controls found abnormalities in cytoskeletal remodeling and the oxidative stress response in patient-derived samples [13]. Similarly, cell proliferation and expression of ion channels were found to be abnormal in NPCs derived from individuals with bipolar disorder [14].

We demonstrate here that *CYFIP1* plays a critical role in cytoskeletal remodeling in human NPCs, as we observe dysregulation of cytoskeleton-related genes, reduced levels of WAVE1/2, and diminished F-actin following knockdown. *CYFIP1* knockdown cells tended to be bigger and had significantly larger nuclei as well. Moreover, reduced *CYFIP1* also disrupted normal expression of fragile X mental retardation protein (FMRP) targets and postsynaptic density (PSD) genes, each previously implicated in schizophrenia [15–17]. Providing direct support for involvement of *CYFIP1* in BP1-2 mediated neurodevelopmental disease (NDD), we observed marked dysregulation of schizophrenia and epilepsy risk genes, disorders associated with the deletion. Mirroring the clinical variability associated with BP1-2 deletions, the presence and magnitude of these disease effects varied between donor lines. Effects were also highly specific in that enrichment was not seen for genes implicated in complex diseases or traits unrelated to the BP1-2 deletion. We also identified novel candidate disease genes among a subset of altered mRNAs that are unexpectedly similar to known epilepsy genes. The *in vitro* model described here will enable identification of compounds that normalize BP1-2 disease-related endpoints and as such represents an important next step towards personalized treatment of deletion carriers.

## Results

### Generation of human neural progenitor cells with reduced *CYFIP1* expression

**Fig 1:**
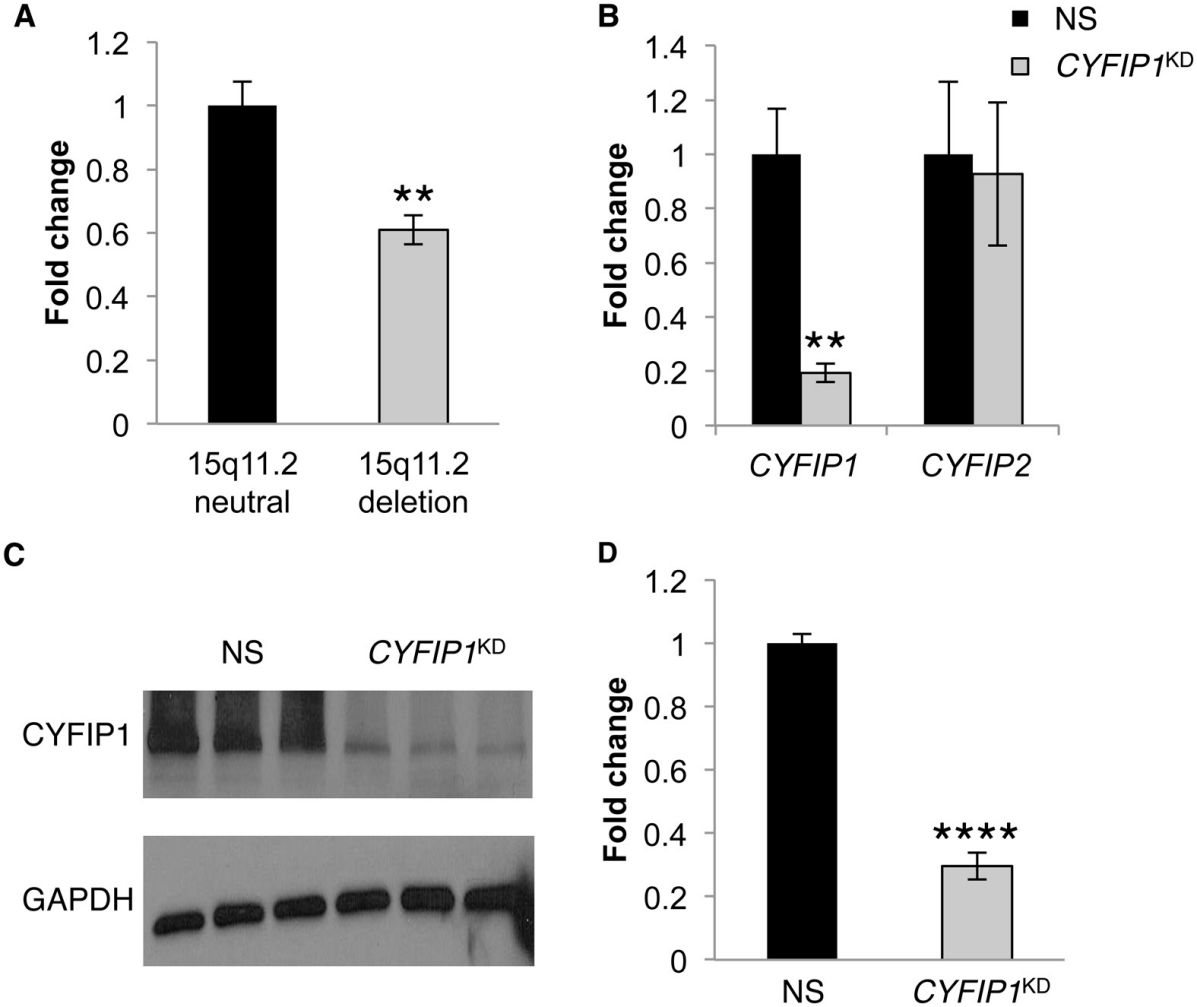
Similar to lymphoblastoid cell lines from individuals with BP1-2 deletions, *CYFIP1* levels are reduced following shRNA knockdown in BP1-2 copy number neutral human neural progenitor cells (NPCs). (**A**) qPCR shows that relative to BP1-2 neutral controls (n = 7), *CYFIP1* mRNA levels are reduced in lymphoblastoid cell lines from deletion carriers (n = 7). (**B**) qPCR on BP1-2 copy number neutral NPCs shows that relative to non-silencing shRNA transduced cells (NS, black), transduction with an shRNA targeted against *CYFIP1* (*CYFIP1*^KD^, grey) results in a reduction of *CYFIP1* but not *CYFIP2* mRNA (n = 6 per group). (**C, D**) Relative to control NPCs, western blot shows that *CYFIP1*^KD^ results in a reduction of CYFIP1 protein (n = 15 per group; 3 or more samples from each of 3 separate transductions). One-tailed Student’s t-test, **p≤ 0.01; ****p≤0.0001.

We sought to model, in a human system, the impact of reduced *CYFIP1* expression with regard to BP1-2-related NDDs. To validate the model, we first confirmed in lymphoblastoid cell lines from BP1-2 deletion carriers that *CYFIP1* mRNA levels were indeed significantly reduced relative to cell lines from BP1-2 copy number neutral individuals (39% reduction; p = 1.4 x 10^−3^; Fig 1A). We next differentiated NPCs from previously characterized human iPSC lines [18–23], and transduced cells with a lentivirus carrying either a non-silencing control shRNA (NS) or an shRNA targeting *CYFIP1* (*CYFIP1*^KD^). To study *CYFIP1*-specific contributions, we limited these and subsequent experiments to lines prepared from BP1-2 copy number neutral male adults with no history of NDDs. We determined that *CYFIP1* levels were reduced significantly in response to knockdown (81% reduction; p = 2.2x 10^−3^; Fig 1B). The extent of *CYFIP1* reduction in our model system falls within the range of what is observed in neural cells derived from BP1-2 deletion carriers [12,24]. mRNA levels of the closely related *CYFIP2* gene were indistinguishable between NS and *CYFIP1*^KD^ NPCs. Paralleling *CYFIP1* mRNA levels, quantification of CYFIP1 protein levels showed a significant reduction in *CYFIP1*^KD^ NPCs relative to NS controls (70% reduction; p = 4.4 x 10^−14^; Fig 1C and 1D).

### Transcriptional profiling in *CYFIP1*^KD^ NPCs identifies hundreds of dysregulated genes

**Fig 2:**
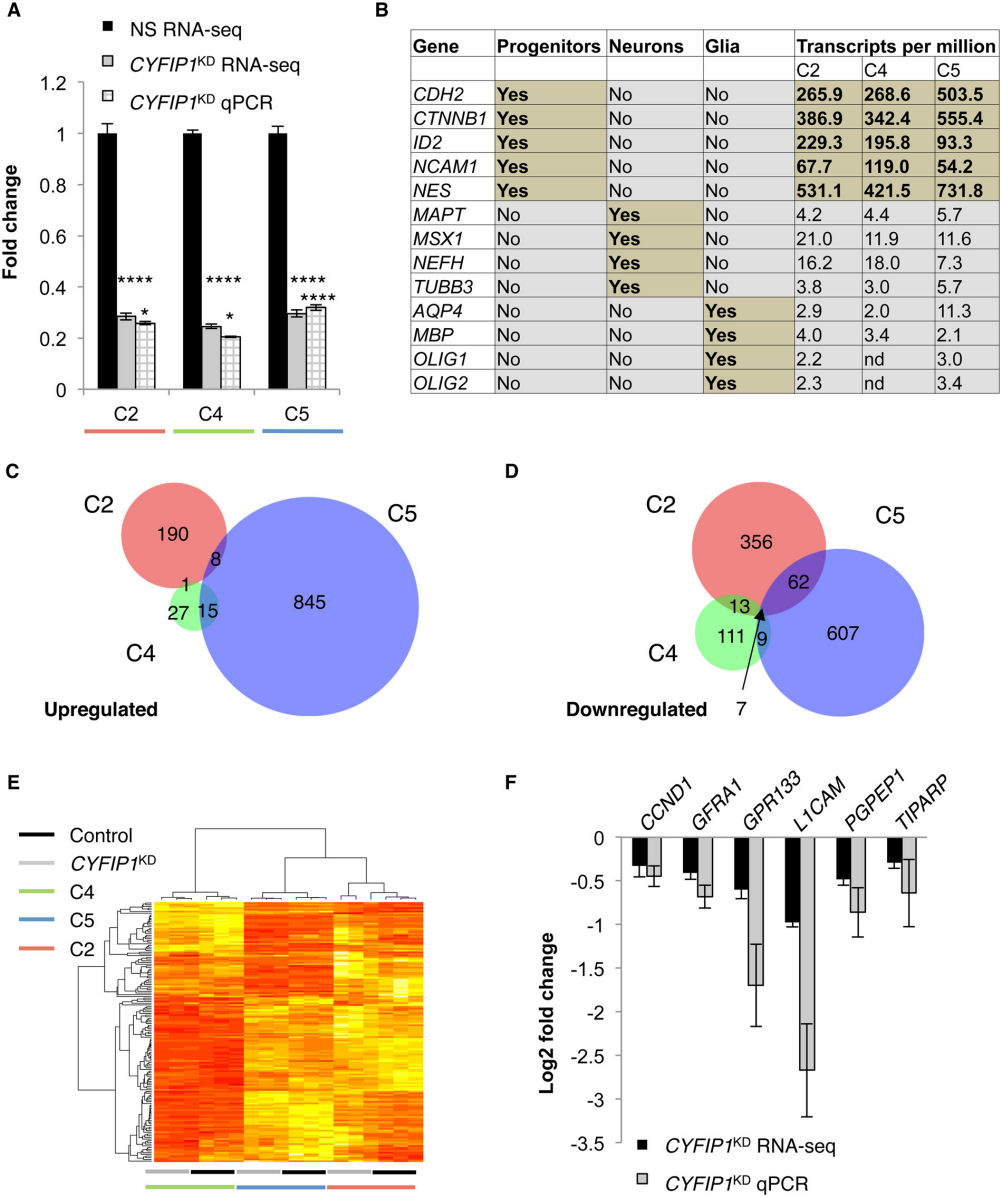
*CYFIP1* knockdown results in dysregulation of hundreds of genes in human neural progenitor cells (NPCs). (**A**) RNA-seq and qPCR show that compared to non-silencing control transduced NPC lines (NS, black), cells transduced with an shRNA targeting *CYFIP1* (*CYFIP1*^KD^, grey and hatched) show a significant reduction in *CYFIP1* mRNA in lines from three different BP1-2 copy number neutral individuals (C2, red; C4, green; C5, blue, n = 3 per group throughout). P-values for RNA-seq analysis- DESeq2; qPCR- one-tailed Student’s t-test; *p≤ 0.05; ****p≤0.0001. (**B**) RNA-seq shows that mRNAs for NPC markers are expressed 10 to 100-fold higher than mRNAs for neuronal and glial markers. TPM values are averaged across NS and *CYFIP1*^KD^ samples. (**C**, **D**) Only a handful of genes are similarly dysregulated in response to *CYFIP1* knockdown across all three NPC lines evaluated. (**E**) Unsupervised hierarchical clustering of genes showing nominal differential expression (p<0.05) points to marked between-line differences. (**F**) RNA-seq and qPCR on mRNA from control and *CYFIP1*^KD^ NPCs (*CYFIP1*^KD^ RNA-seq, black; *CYFIP1*^KD^ qPCR, grey) show reduced levels of genes downregulated in all three cell lines. Abbreviations: *CCND1*- cyclin D1, *GFRA1*- GDNF family receptor alpha-1, *GPR133*- G-protein coupled receptor 133, *L1CAM*- L1 cell adhesion molecule, *PGPEP1*- pyroglutamyl-peptidase I, *TIPARP*- TCDD-inducible poly(ADP-ribose) polymerase.

To gain insight into the molecular effects of reduced *CYFIP1* expression, we performed RNA-seq on NS and *CYFIP1*^KD^ NPCs prepared from three different iPSC lines (C2, C4, and C5). Because each line was generated from a different donor, we reasoned that this experimental design would allow us to evaluate line-dependent and line-independent effects. For each line, RNA from six independent transductions (three *CYFIP1*^KD^ and three NS) was sequenced, and an average of 23.2 million properly mapped pairs was obtained (S1 Table). For each donor line, the average of the three *CYFIP1*^KD^ samples was compared to the average of the three isogenic NS samples. RNA-seq and qPCR validation showed that *CYFIP1* was similarly reduced in NPCs from all three lines (Fig 2A). Confirming that the cells we transduced were indeed in a progenitor state, all lines showed high levels of neural progenitor markers and much lower expression of neuronal and glial markers (Fig 2B).

We next sought to identify genes sensitive to reduced levels of *CYFIP1* (differentially expressed genes, DEGs). Using a stringent cutoff (transcripts per million (TPM)>1 in NS samples and FDR-adjusted p<0.05), 2212 unique genes were found to be dysregulated in NPCs from one or more lines, with 637 DEGs in C2, 183 DEGs in C4, and 1553 DEGs in C5 (Fig 2C and 2D, S2 and S3 Tables). Unsupervised hierarchical clustering of genes showing nominal differential expression (p<0.05) in all three lines (n = 153) revealed that samples clustered first by line, and then by knockdown status (Fig 2E). Using the more stringent cutoff, only seven DEGs (including *CYFIP1*) showed dysregulation in all three lines. Excluding *CYFIP1*, evaluation of the six remaining genes altered between all three lines (*CCND1*, *GFRA1*, *GPR133*, *L1CAM*, *PGPEP1*, *TIPARP)* in line C2 by qPCR confirmed DEG status for each (Fig 2F).

### Genes involved in M phase of cell cycle and cytoskeletal remodeling are overrepresented among dysregulated mRNAs

**Fig 3:**
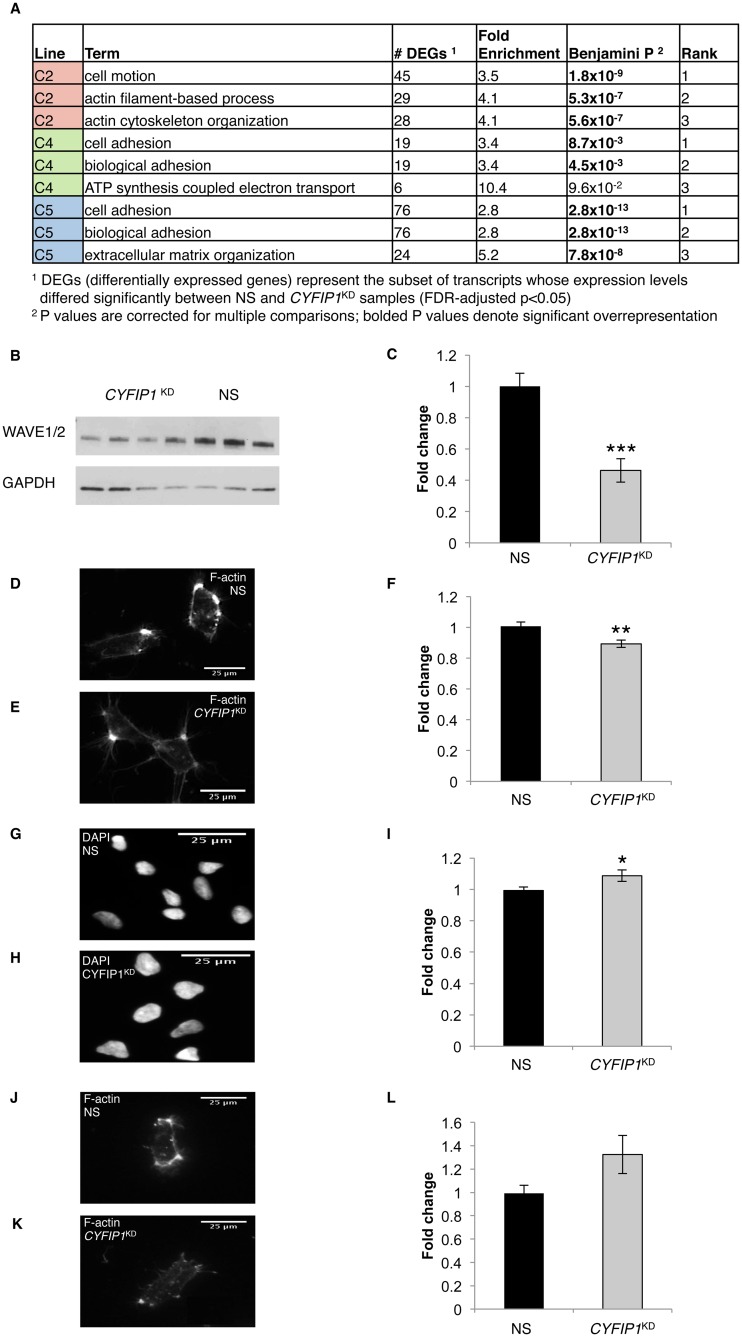
Dysregulation of cytoskeletal remodeling in response to *CYFIP1* knockdown in human neural progenitor cells (NPCs). (**A**) Genes involved in cytoskeletal remodeling are overrepresented among transcripts downregulated in response to *CYFIP1* knockdown. (**B, C**) Western blot shows that relative to non-silencing shRNA transduced NPCs (NS, black), transduction with an shRNA targeted against *CYFIP1* (*CYFIP1*^KD^, grey) results in a significant 54% reduction of WAVE1/2 protein levels. n = 10 per group. (**D, E, F**) Phalloidin staining shows that F-actin levels are significantly reduced (11% reduction) in *CYFIP1*^KD^ NPCs relative to control cells. n = 12 wells per group. (**G, H, I**) Nuclear area, quantified by DAPI, is significantly increased by 9% in *CYFIP1*^KD^ NPCs relative to control cells. n = 12 wells per group. (**J, K, L**) Cell area, captured by phalloidin staining, is nominally greater in *CYFIP1*^KD^ NPCs relative to control cells (34% increase). n = 12 wells per group. Two-tailed Student’s t-test, *p<0.05 **p≤0.01 ***p≤0.001.

We next reasoned that while the majority of genes dysregulated in NPCs prepared from each of the three lines differed, the underlying signaling pathways altered in each might overlap. To test this hypothesis, we carried out a series of gene ontology (GO) analyses on the upregulated and downregulated DEGs from each line. Our analyses identified an enrichment of genes involved in M phase of the cell cycle in lines C4 and C5 (Table 1). Also consistent with dysregulation of shared processes was the overrepresentation of cytoskeletal-related GO terms among downregulated genes from each of the three lines (Fig 3A). C2 was enriched for cell motion and other actin based processes while C4 and C5 were enriched for cell adhesion, another cytoskeletal based function [25,26]. Together these results demonstrate that while the particular genes dysregulated in response to *CYFIP1*^KD^ differed between NPCs from each line, similar processes were impacted in response to reduced expression. Furthermore, these studies are the first to show that reduced levels of *CYFIP1* affect transcriptional control of cytoskeletal genes.

**Table 1:** Genes involved in M phase of cell cycle are overrepresented among transcripts upregulated in response to *CYFIP1* knockdown in human neural progenitors.

Line	Term	# DEGs^1^	Fold Enrichment	Benjamini P^2^	Rank
C2	biological adhesion	16	2.4	7.1x10^-1^	1
C2	vasodilation	3	11.2	8.7x10^-1^	2
C2	positive regulation of cell motion	5	4.4	8.7x10^-1^	3
C4	cell division	10	10.4	**1.4x10**^**-4**^	1
C4	M phase	9	8.8	**1.5x10**^**-3**^	2
C4	cell cycle	12	4.8	**2.8x10**^**-3**^	3
C5	M phase	113	5.7	**3.7x10**^**-53**^	1
C5	cell cycle	173	3.6	**1.7x10**^**-52**^	2
C5	cell cycle phase	124	4.9	**4.7x10**^**-51**^	3

^1^ DEGs (differentially expressed genes) represent the subset of transcripts whose expression levels differed significantly between NS and *CYFIP1*^KD^ samples (FDR-adjusted p<0.05)

^2^ P values are corrected for multiple comparisons; bolded P values denote significant overrepresentation

### Functional studies corroborate molecular findings for aberrant cytoskeletal remodeling in *CYFIP1*^KD^ NPCs

We next aimed to validate a subset of these molecular findings functionally, focusing on cytoskeletal remodeling. Although CYFIP1 is well known to play a role in cytoskeletal remodeling in neurons [6,7,9,10,27], its role in NPCs has received less attention. To ensure that our findings here were line-independent, this work was carried out in NPCs prepared from iPSCs that were not used in our RNA-seq experiments (see Materials and Methods). We first measured WAVE1/2 protein levels in *CYFIP1*^KD^ and NS NPCs. CYFIP1 and WAVE proteins are members of the Wave Regulatory Complex (WRC), a key mediator of cytoskeletal dynamics. Previous studies have shown that inactivation of individual components of the WRC leads to reduced expression of the other WRC members [9,28]. Moreover, neural rosettes prepared from BP1-2 deletion carrier iPSCs show diminished WAVE2 levels as a consequence of reduced *CYFIP1* expression [12]. Consistent with what is seen in neural cells derived from BP1-2 deletion carriers, examination of WAVE1/2 protein levels in our system showed a significant reduction in *CYFIP1*^KD^ NPCs relative to controls (54% reduction; p = 1.4 x 10^−4^; Fig 3B and 3C). Because the WRC is a critical regulator of actin polymerization, we next stained *CYFIP1*^KD^ and NS NPCs with phalloidin to permit quantification of F-actin levels. These experiments showed that *CYFIP1*^KD^ resulted in a significant reduction of F-actin levels (11% reduction; p = 5.0 x 10^−3^; Fig 3D–3F). In the course of these experiments we also determined that nuclear size was significantly increased in *CYFIP1*^KD^ NPCs when compared to NS cells using a DAPI stain (9% increase; p = 3.4 x 10^−2^ Fig 3G–3I). *CYFIP1*^KD^ NPCs also tended to be larger than NS NPCs although this apparent difference was not significant (34% increase; p = 0.08; Fig 3J–3L). These findings corroborate RNA-seq results and underscore a prominent role for *CYFIP1* in cytoskeletal regulation in NPCs.

### *CYFIP1*^KD^ in NPCs results in transcriptional dysregulation of FMRP targets and postsynaptic density genes

To further explore how reduced levels of *CYFIP1* may impact NPC function, we looked for overrepresentation of FMRP targets and PSD genes (S4 Table) among DEGs. CYFIP1 is known to physically interact with FMRP to repress translation of FMRP-associated mRNAs [5,6]. Alterations in *CYFIP1* levels have also been shown to cause changes to dendritic morphology in differentiated neurons, where proteins of PSD genes localize [6,7,10]. Our analyses determined that FRMP targets were significantly enriched among DEGs across all lines (C2: p = 6.1x10^-3^, Fold Enrichment (FE) = 1.6; C4: p = 1.5x10^-3^, FE = 2.2; C5: p = 1.7x10^-6^, FE = 1.6; Table 2 and S3 Table), showing for the first time that *CYFIP1* exerts not only a translational control over FMRP targets, but a transcriptional control as well. As with FMRP targets, enrichment analyses revealed a line-independent overrepresentation of PSD genes among DEGs (C2: p = 9.9x10^-7^, FE = 2.2; C4: p = 1.0x10^-3^, FE = 2.5; C5 p = 1.6x10^-7^, FE = 1.8; Table 2 and S3 Table). Additional GO analyses of FMRP and PSD-related DEGs showed that PSD genes dysregulated in response to *CYFIP1*^KD^ were significantly enriched for genes involved in cytoskeleton-based processes (S5 Table), providing a satisfying explanation for this finding. Given that candidate mutations in both FMRP targets and PSD genes are present at a significantly greater frequency in individuals with schizophrenia compared to typically developing controls [15–17], these results suggest that reduced levels of *CYFIP1* might increase risk for schizophrenia at least in part through dysregulation of one or both of these functional networks. Results also support the idea that abnormalities in NPCs might in some cases predict neuronal dysfunction.

**Table 2:** Dysregulation of FMRP targets and post-synaptic density genes in response to *CYFIP1* knockdown in human neural progenitor cells.

Gene Group	Line	# EGs^1^	# DEGs^2^	Fold Enrichment	Fisher's Exact P^3^
FMRP	C2	819	41	1.6	**6.1x10**^**-3**^
PSD	C2	611	44	2.2	**9.9x10**^**-7**^
FMRP	C4	808	18	2.2	**1.5x10**^**-3**^
PSD	C4	596	15	2.5	**1.0x10**^**-3**^
FMRP	C5	814	106	1.6	**1.7x10**^**-6**^
PSD	C5	608	88	1.8	**1.6x10**^**-7**^

^1^ EGs (expressed genes) correspond to the subset of transcripts within a given category (ex. FMRP targets) for which the average transcript per million (TPM) count was greater than 1 in NS control NPCs

^2^ DEGs (differentially expressed genes) represent the subset of transcripts within a given category (ex. FMRP targets) whose expression levels differed significantly between NS and *CYFIP1*^KD^ samples (FDR-adjusted p<0.05)

^3^ Bolded P values denote significant overrepresentation

### *CYFIP1*^KD^ results in dysregulation of genes implicated in schizophrenia and epilepsy, but not diseases or traits unrelated to BP1-2 deletions

BP1-2 deletions are well established to increase risk for both schizophrenia and epilepsy [1–3]. Clinical presentation, however, is highly variable and many carriers do not meet diagnostic criteria for any NDD [29,30]. Given evidence for dysregulation of disease-related pathways by *CYFIP1*^KD^, we sought to determine if reduced levels of *CYFIP1* in NPCs might alter the expression of BP1-2 deletion-related disease gene networks. To test this hypothesis, we identified lists of genes associated with schizophrenia and epilepsy (S4 Table) and looked for overrepresentation among *CYFIP1*^KD^ DEGs (Table 3 and S3 Table). Overrepresentation of schizophrenia risk genes was seen in C2 (p = 4.6 x10^-2^, FE = 1.5) and C5 (p = 4.9x10^-4^, FE = 1.5) but not C4 (p = 1.0, FE = 1.0). With regard to epilepsy, overrepresentation of *CYFIP1*^KD^ DEGs was observed in two of three lines, although the identity of these lines differed from those in which the schizophrenia effects were observed. Overrepresentation here was seen in C4 (p = 3.2x10^-8^, FE = 10.7) and C5 (p = 6.0x10^-3^, FE = 2.0) but not C2 (p = 0.58, FE = 1.2). Effects were not associated with either the number of DEGs per line or efficiency of *CYFIP1* knockdown. These data demonstrate that reduction of *CYFIP1* levels can alter BP1-2 deletion-related disease gene networks, but in contrast to dysregulation of cytoskeleton genes, FMRP targets, and PSD genes, the presence and magnitude of these disease effects were variable, much like clinical outcomes in deletion carriers.

**Table 3:** Genes implicated in schizophrenia and epilepsy, but not conditions unrelated to BP1-2 deletions, are overrepresented among *CYFIP1* knockdown dysregulated mRNAs.

Disease/Trait^1^	Line	#EGs^2^	#DEGs^3^	Fold Enrichment	Fisher's Exact P^4^
Schizophrenia	C2	536	26	1.5	**4.6x10**^**-2**^
Epilepsy	C2	104	4	1.2	0.58
Rheumatoid Arthritis	C2	84	4	1.5	0.35
Autism	C2	286	14	1.5	0.13
Schizophrenia	C4	512	5	1.0	1.0
Epilepsy	C4	93	10	10.7	**3.2x10**^**-8**^
Rheumatoid Arthritis	C4	80	1	1.2	0.55
Autism	C4	275	4	1.5	0.36
Schizophrenia	C5	525	66	1.5	**4.9x10**^**-4**^
Epilepsy	C5	104	17	2.0	**6.0x10**^**-3**^
Rheumatoid Arthritis	C5	79	6	0.9	1.0
Autism	C5	279	32	1.4	0.06

^1^ Additional traits examined: breast cancer, type II diabetes, Crohn’s disease, body mass index, blood pressure, height, and intellectual disability (results in S6 Table)

^2^ EGs (expressed genes) correspond to the subset of transcripts within a given category (ex. schizophrenia-associated genes) for which the average transcript per million (TPM) count was greater than 1 in NS control NPCs

^3^ DEGs (differentially expressed genes) represent the subset of expressed genes within a given category (ex. schizophrenia-associated genes) whose expression levels differed significantly between NS and *CYFIP1*^KD^ samples (FDR-adjusted p<0.05)

^4^ Bolded P values denote significant overrepresentation

To evaluate the specificity of these effects, we assembled gene lists for diseases and traits with no known association to BP1-2 deletion status (S4 Table). As outlined in Table 3 and S6 Table, in contrast to findings for schizophrenia and epilepsy where strong effects were seen, nothing comparable was observed for: rheumatoid arthritis (p_min_ = 0.35, FE = 1.5 for C2), breast cancer (p_min_ = 0.13, FE = 1.6 for C5), type II diabetes (p_min_ = 0.03, FE = 1.8 for C5), Crohn's disease (p_min_ = 0.01, FE = 2.6 for C2), body mass index (p_min_ = 0.24, FE = 0.4 for C5), or blood pressure (p_min_ = 0.16, FE = 1.9 for C2). An effect for height was observed in one line (p_min_ = 1.5x10^-3^, FE = 1.7 for C5). Additionally, while case-only studies have suggested relationships between deletion status and both autism and intellectual disability, these results are confounded by ascertainment bias, and no clear effects have been seen in population based studies [4,31–33]. Consistent with the absence of a relationship, we did not see an enrichment for genes implicated in autism (p_min_ = 0.06, FE = 1.4 for C5) or intellectual disability (p_min_ = 0.14, FE = 1.4 for C5). Finally, if considered together, a disproportionate number of nominally significant effects are from analyses for schizophrenia and epilepsy as opposed to diseases and traits with no evidence for an association with deletion status (4/6 vs. 3/27; p = 0.01, two-sided Fisher’s exact test).

### Interrogation of schizophrenia and epilepsy genes elucidates mechanisms and identifies novel disease candidates

To gain a better understanding of what disease genes that are sensitive to reduced levels of *CYFIP1* do functionally, we performed additional GO analyses. With regard to schizophrenia, we reasoned that such analyses might provide additional mechanistic insights beyond possible contributions from FRMP targets and PSD genes. However, this was not the case; analysis of the schizophrenia-associated genes included within our list of *CYFIP1*^KD^ DEGs did not reveal a significant enrichment of any GO terms (S7 Table). Meanwhile, GO analysis of epilepsy-related *CYFIP1*^KD^ DEGs showed a strong enrichment for genes involved in synaptic transmission (Table 4). A closer examination of these genes revealed that they primarily code for ion channels such as *CACNA1H*, *KCNA1*, and *SCN1A*, providing additional support for the idea that signs of neuronal dysfunction may be present at the NPC stage. GO analyses also found evidence for genes involved in mitochondrial electron transport (Table 4).

**Table 4:** Epilepsy genes dysregulated in response to *CYFIP1* knockdown are involved in synaptic transmission.

Term	# DEGs^1^	Fold Enrichment	Benjamini P^2^	Rank
transmission of nerve impulse	11	11.8	**2.2x10**^**-6**^	1
synaptic transmission	10	12	**7.6x10**^**-6**^	2
cell-cell signaling	10	8.7	**9.5x10**^**-5**^	3
negative regulation of cyclase activity	6	27.7	**1.2x10**^**-4**^	4
negative regulation of lyase activity	6	27.7	**1.2x10**^**-4**^	5
mitochondrial electron transport, NADH to ubiquinone	4	40	**3.3x10**^**-3**^	20
multicellular organismal response to stress	4	34.3	**5.3x10**^**-3**^	21
negative regulation of catalytic activity	7	7.8	**5.8x10**^**-3**^	22
mitochondrial ATP synthesis coupled electron transport	4	26.7	**1.1x10**^**-2**^	23
ATP synthesis coupled electron transport	4	26.7	**1.1x10**^**-2**^	24
respiratory electron transport chain	4	24	**1.4x10**^**-2**^	25

^1^ DEGs (differentially expressed genes) represent the subset of transcripts that are epilepsy-associated genes whose expression levels differed significantly between NS and *CYFIP1*^KD^ samples (FDR-adjusted p<0.05)

^2^ P values are corrected for multiple comparisons; bolded P values denote significant overrepresentation

Given the marked overrepresentation of well-established epilepsy genes among *CYFIP1*^*KD*^ DEGs, we reasoned that a subset of these might represent novel disease candidates. Employing the same epilepsy gene list used in our overrepresentation analysis as a training set, we used ToppGene [34] to identify candidates. The top 10 genes from each line (Table 5; complete output- S8 Table), corresponding to 28 unique genes in total, showed unexpected similarity to known disease genes (C2: p<3.2x10^-5^; C4: p<1.1x10^-4^; C5: p = 4.0x10^-7^). Two genes, *CLIC4* and *LOC102724434*, appeared in the top 10 ranked genes for both C2 and C5. Validating the concept that these 28 epilepsy-like *CYFIP1*^KD^ DEGs are good disease gene candidates, a post-analysis literature review determined that *CACNA1C* and *MT-ATP6*, although not included in our training set, are in fact known to cause epilepsy [35,36]. Likewise, 12/28 genes have been implicated in epilepsy or have a close family member established to increase risk including *SCN2B*, *CALM1*, *CALM2*, and *APP* [37–46]. These 12 genes, and the additional 14 not previously linked to disease, represent new epilepsy candidates that can be evaluated in the near term through sequencing studies of patients and controls.

**Table 5:** A subset of genes dysregulated as a result of *CYFIP1* knockdown show striking similarity to known epilepsy genes, and as such are strong candidate disease genes.

Line	Gene	Overall P^1^	Rank	Known^2^	Prior Evidence^3^
C2	*CALM1*	9.1x10^-7^	1		✓
C2	*SCN2B*	1.7x10^-6^	2		✓
C2	*CLIC4*	2.1x10^-6^	3		✓
C2	*CACNA2D3*	6.2x10^-6^	4		✓
C2	*SNCA*	9.2x10^-6^	5		✓
C2	*LOC102724434*	3.2x10^-5^	6		
C2	*LOC101927943*	3.2x10^-5^	7		
C2	*LOC100505504*	3.2x10^-5^	8		
C2	*LOC100505501*	3.2x10^-5^	9		
C2	*FLJ43879*	3.2x10^-5^	10		
C4	*APP*	4.4x10^-7^	1		✓
C4	*MT-ATP8*	1.6x10^-5^	2		✓
C4	*MT-ND2*	3.9x10^-5^	3		✓
C4	*SLC8A1*	5.4x10^-5^	4		
C4	*SLC1A4*	6.7x10^-5^	5		✓
C4	*MT-ATP6*	7.1x10^-5^	6	PMID:2137962	
C4	*MT-RNR1*	8.3x10^-5^	7		
C4	*SLC38A1*	1.0x10^-4^	8		✓
C4	*STX12*	1.0x10^-4^	9		
C4	*FEZ1*	1.1x10^-4^	10		
C5	*CACNA1C*	1.9x10^-9^	1	PMID: 15454078	
C5	*CLIC4*	6.2x10^-8^	2		✓
C5	*CALM2*	6.8x10^-8^	3		✓
C5	*LOC729652*	1.3x10^-7^	4		
C5	*RNA5-8SP6*	1.3x10^-7^	5		
C5	*LOC102724434*	1.3x10^-7^	6		
C5	*LINC01001*	1.3x10^-7^	7		
C5	*MIR22HG*	3.8x10^-7^	8		
C5	*GNG2*	3.9x10^-7^	9		✓
C5	*LOC101929680*	4.0x10^-7^	10		

^1^ per ToppGene

^2^ These genes were not included in our training set but found through literature review after the completion of analyses to be causal for epilepsy

^3^ A post-analysis literature review identified evidence implicating these genes in epilepsy

## Discussion

We present a novel human model for studying early developmental abnormalities associated with BP1-2 deletion mediated disease risk. Results from molecular and cellular analyses support an important role for *CYFIP1* in cytoskeletal regulation in NPCs and provide evidence for transcriptional regulation of these processes. Although the specific genes dysregulated in each line differed, the underlying signaling pathways perturbed were largely shared across lines. Similarly, RNA-seq data show that *CYFIP1*^KD^ resulted in line-independent overrepresentation of both FMRP-targets and PSD genes among DEGs.

*CYFIP1*^KD^ also resulted in a significant dysregulation of BP1-2 disease gene networks, but not in an overrepresentation of genes associated with randomly selected disorders or traits. Like clinical presentation in deletion carriers, however, clear between-line differences were evident. A variety of explanations could account for the line—to—line differences we observed, but like BP1-2 mediated risk for NDD, a multifactorial explanation is most probable. For example, it could be that the genomes of the individuals that our iPSC lines were generated from harbor additional genetic risk factors that act in an additive fashion to alter gene expression of disease-associated genes. Consistent with such a mechanism, children who harbor a BP1-2 deletion and a second large CNV are more likely to show developmental delay than those who carry only the deletion [47]. Also relevant is that behavioral deficits seen in mice heterozygous for a mutation in *FMR1* are highly dependent on genetic background [48–50]. Alternatively, between-line differences may reflect the effects of environmental exposures unique to individual donors. It could also be the case that differences are the result of random genetic or epigenetic events that arose during reprogramming or subtle procedural differences during cell culture. This question will be clarified through characterization of additional clones generated from the same donors as the lines studied here and through study of additional clones from unrelated individuals.

Nevertheless, the overarching approach of utilizing materials from multiple individuals and focusing on differences as well as similarities is likely to be of great value. So far as we know, this is the first study to use iPSC derived cells from multiple healthy individuals to study NDDs. Previous studies that used an approach similar to ours looked at materials from only a single donor [23,51,52]. As our data reveal, results can vary between lines, and so caution must be exercised when interpreting results from studies that only use one line for their experiments. This is particularly true with regard towards translational efforts for risk factors like BP1-2 deletions where inter-individual variability is a key feature.

In terms of mechanisms, dysregulation of FMRP targets and PSD genes is intriguing given previous studies that suggest both pathways are related to schizophrenia risk [15–17]. Because FMRP and PSD effects were observed in all lines, including line C4 where we did not observe an overrepresentation of schizophrenia candidate genes, alteration of these signaling pathways cannot fully explain the observed schizophrenia effect. Additional work will be required to identify additional contributory factors. Meanwhile, genes involved in regulation of M phase might be relevant to understanding dysregulation of epilepsy gene networks; such transcripts were overrepresented among DEGs from C4 and C5, the same two lines in which an overrepresentation of epilepsy genes was observed.

PSD and synaptic transmission effects were initially surprising given that these gene networks are typically associated with differentiated neurons. Subsequent analyses, however, highlighting a large number of cytoskeleton-related genes within the set of PSD genes dysregulated by *CYFIP1*^KD^ provide some clarity here. Results suggest that disease-related biological signatures are evident long before neuronal differentiation and support the notion that early development insults may confer risk for disease. The study of iPSCs derived neurons has emerged as a popular means of modeling disease [53–56], but less attention has been given to NPCs. Support for the use of NPCs has come from studies of schizophrenia, bipolar disorder, and Rett Syndrome [13,14,57–59]. For example, Topol *et al*. discovered altered WNT signaling in NPCs derived from schizophrenia patients, a pathway previously known to be dysregulated in post-mortem brain of individuals with schizophrenia and thought to be a target of many antipsychotic drugs [60,61]. Our findings provide further support for the idea that NPCs can be useful in studying NDD risk and may in some cases circumvent the time and cost associated with study of differentiated neurons. However, it will be interesting to determine the effects of *CYFIP1* knockdown on differentiated neurons and the differentiation process in future experiments.

Lastly, although dysregulation of *CYFIP1* alone may not account for all aspects of BP1-2 associated risk, our data point to the importance of this gene. For example, results from our *CYFIP1* knockdown model are consistent with what is seen in neural cells derived from BP1-2 deletion carriers. Additionally, in each of the three lines we evaluated, *CYFIP1*^KD^ resulted in the dysregulation of one or more disease gene networks. In the face of these data, high-throughput drug screening in our model system could be useful in the identification of candidate compounds that could be evaluated in deletion carriers.

## Materials and Methods

### Ethics Statement

Subjects who provided us with materials to generate lymphoblastoid cell lines were enrolled through Einstein and provided written informed consent. For minors enrolled in this study, informed written consent was obtained via a parent. Subjects from which iPSCs were generated were recruited from two settings; Einstein and the National Institutes of Mental Health (NIMH), Child Psychiatry Branch. For the NIMH subjects, all participants provided written assent or consent. These studies, consent forms, and procedures for obtaining informed consent were approved by the Einstein or the NIMH Institutional Review Board.

### Lymphoblastoid Cell Lines

Eight lymphoblastoid cell lines, four from BP1-2 neutral subjects and four from deletion carriers, were obtained from the Simons Simplex Collection [62]. An additional six lines, three from neutral subjects and three from deletion carriers, were generated at Einstein. Peripheral blood was collected from subjects and lymphoblastoid cells were immortalized by Epstein Barr virus transformation. See S9 Table regarding clinical information for subjects.

### BP1-2 Copy Number Variant Genotyping

Before experimentation, a TaqMan assay Hs01476346_cn (Life Technologies) was used to determine copy number at the BP1-2 region in all lymphoblastoid and NPC lines used. Assays were performed in triplicate with TaqMan Gene Expression Master Mix (Life Technologies) following the standard TaqMan Copy Number Assay protocol. An RNase P probe (Cat# 4403326, Life Technologies) was used as a reference. Copy number variants were called using Copy Caller Software (v2.0, Life Technologies).

### iPSC Lines and NPC Differentiation

iPSC lines were generated from fibroblast cells taken from skin biopsies of healthy adults with no reported NDDs. iPSC lines used in this study have been described previously [18–23]. The lines used for RNA-seq in this study were: 553.C2 (C2), iPS5.C4 (C4), 690.C5 (C5). These lines were generated from a 31 year old Caucasian male, a 33 year old Caucasian male, and a 27 year old Caucasian male, respectively. Lines iPS2.C4 and iPS6.C4, used for functional studies, were generated from a 57 year old Caucasian male and a 46 year old Caucasian male, respectively. Reprogramming was carried out by nucleofection of non-integrating plasmids containing *OCT4*, *SOX2*, *KLF4*, *L-MYC*, *LIN28*, and a *p53* shRNA vector (Cat# 27077, Addgene). All lines have normal karyotypes, express stem cell markers, and can be differentiated into all three germ layers. Differentiation into NPCs was performed was as previously described [18–20,22,23]. All lines were confirmed by Taqman assay Hs01476346_cn (see above) to not carry a BP1-2 deletion or duplication.

### Cell Culture and Lentiviral Transduction

All cultures were maintained in humidified incubators at 37°C and 5% CO_2_ in air. Lymphoblastoid cell lines were cultured in RPMI 1640 with 15% heat inactivated fetal bovine serum, 1% Glutamate, and 1% penicillin/streptomycin (pen/strep) (Life Technologies). NPCs were maintained on polyornithine/laminin coated plates and were fed every other day with NBF media (DMEM/F12 supplemented with 0.5% N-2, 0.5% B-27, and 1% pen/strep (Life Technologies)) and fresh 20ng/ml FGF2. FGF2 from Gemini was used for all RNA-seq studies. For functional studies FGF2 from Gemini or R&D Systems was used.

NPCs were transduced with GIPZ lentivirus carrying either an shRNA targeting *CYFIP1* (Oligo ID- V2LHS_258463, sense sequence- GCAAAGATGAGATTATTAA, Open Biosystems) or a non-silencing control (Cat#- RHS 4346, sense sequence- CTCGCTTGGGCGAGAGTAA, Open Biosystems). Lentiviruses were made in HEK293T cells using 2^nd^ generation packaging plasmids. Transductions were carried out when NPCs were 50% confluent. Briefly, 1.0–1.5x10^6^ viral particles were added to NPCs in transduction media (NBF media, 1 μg/ml polybrene (Santa Cruz), and 20ng/ml FGF2). Twenty-four hours after transduction, media was replaced with fresh NBF and fresh FGF2. To select for transduced cells, media was changed again 48 hours later with fresh NBF/FGF2 but with the addition of 0.5 μg/ml puromycin (Santa Cruz). This selection media was replaced every other day.

### RNA-seq

When NPCs were 100% confluent, total RNA was isolated using a miRNAeasy Kit (Qiagen) according to the manufacturer’s protocol. An additional DNaseI digestion step was performed to ensure that samples were not contaminated with genomic DNA (Qiagen). RNA quality was assessed via an Agilent bioanalyzer. One hundred bp paired-end RNA-seq was carried out on an Illumina 2000 HiSeq at the JP Sulzberger Columbia Genome Center at Columbia University. The 100 bp paired-end reads were processed as previously described [21]. Briefly, sequence quality for each sample was evaluated with FastQC (http://www.bioinformatics.babraham.ac.uk/projects/fastqc/) (S1 Table). No evidence of adapter contamination was observed and average read quality scores > 30 for all samples. Reads were then aligned with TopHat (v 2.0.4) to the human genome (hg19) [63]. HTSeq (v 0.6.1) [64] was used to obtain read counts for each gene (Ensembl release 74) and DESeq2 utilized to determine differentially expressed genes [65]. Relative expression was measured in transcripts per million (TPM) [66]. Unless otherwise stated, only genes with a TPM>1 in non-silencing control samples and a FDR-adjusted p<0.05 were considered differentially expressed. RNA-seq data has been submitted to the Gene Expression Omnibus (GEO) repository [67] (accession number- GSE70935).

### Quantitative real-time PCR (qPCR)

Total RNA was isolated from cells as above. RNA was reverse-transcribed to cDNA with the SuperScript III First Strand Synthesis System for RT-PCR (Life Technologies). qPCR reactions were performed in triplicate with Power SYBR Green Master Mix (Life Technologies). Genes of interested were normalized to the housekeeping gene, *B2M*, and relative changes were calculated using the Pfaffl method. Primers used for qPCR can be found in S10 Table.

### Gene Ontology Enrichment Analyses

The Database for Annotation, Visualization, and Integrated Discovery (DAVID) (http://david.abcc.ncifcrf.gov/home.jsp) was used for gene ontology analyses [68,69]. Ensembl IDs were used as input. For each line, genes expressed in non-silencing control NPC samples with a TPM>1 were used for background (S2 Table). DEGs used for GO analyses are noted in S3 Table. GO terms with Benjamini p-values <0.05 were considered significantly enriched.

### Western Blot

Cells were homogenized in RIPA lysis buffer containing a protease and phosphatase inhibitor cocktail (Thermo Scientific). Lysates were incubated on ice for 10 min, sonicated for 10 min, and centrifuged at 15,000 x g for 15min at 4°C. Protein concentration was determined using a Pierce BCA Protein Assay Kit (Thermo Scientific). An equal amount of protein for each sample was run on a 4–20% gradient gel (BioRad) and transferred onto a PVDF membrane. Membranes were incubated with primary antibodies overnight at 4°C, incubated with secondary antibodies for 1 hr at room temperature, and visualized with SuperSignal West Dura Chemiluminescent Substrate (Thermo Scientific). Primary antibody dilutions were as follows: rabbit polyclonal anti-CYFIP1 1:1000 (Cat#07–531, Millipore), rabbit polyclonal anti-WAVE1/2 1:1000 (Cat#sc-5557, Santa Cruz), mouse monoclonal anti-GAPDH 1:20000 (Cat#10R-G109a, Fitzgerald).

### Immunocytochemistry and Microscopy

NPCs were grown on polyornithine/laminin coated coverslips. Cells were fixed in 4% formaldehyde for 10 min at room temperature and permeabilized with 0.1% Triton X-100 in PBS for 5 min at room temperature. Coverslips were incubated with a rhodamine phalloidin probe (Life Technologies) at a 1:40 dilution for 1 hour at room temperature. Samples were then mounted with ProLong Gold Antifade with DAPI (Life Technologies). Images were taken on a Zeiss AxioImager at 20x magnification. Images were analyzed for F-actin intensity and nuclear size (area) with Volocity (v 6.3). Cell size (area) was analyzed with ImageJ.

### Gene Lists

FMRP targets and PSD genes, provided in S4 Table, were from Supplementary S2 Table from [70] and Supplementary S2 Table from [71], respectively. Lists of disease and trait-related genes, also provided in S4 Table, were as follows: Schizophrenia- *de novo* hits from [15]; Epilepsy- the union of GeneEpi (http://epilepsy.hardwicklab.org/) and BrainSpan (http://www.brainspan.org) [72]; Rheumatoid Arthritis, Breast Cancer, Type II Diabetes, Crohn’s Disease, Body Mass Index, Blood Pressure, and Height- the NHGRI-EBI Catalog of published genome-wide association studies (https://www.ebi.ac.uk/gwas/) [73]; Autism- the union of categories S through 5 from SFARI Gene (https://gene.sfari.org/autdb/Welcome.do) [74]; Intellectual Disability- Supplementary Table 6 from [75].

### ToppGene

ToppGene (https://toppgene.cchmc.org) was used for prioritization of candidate genes as previously described [34]. The union of genes from GeneEpi (http://epilepsy.hardwicklab.org/) and BrainSpan (http://www.brainspan.org) [72] that was used for our epilepsy enrichment analyses was employed as our training set. Differentially expressed genes from each line were each used as separate test sets. Default cutoffs and gene limits were used for analyses with the addition of Interaction and Gene Family categories.

### Statistical Analysis

Differentially expressed genes were determined by DESeq2 using a cutoff of TPM>1 in non-silencing control samples and an FDR-adjusted p<0.05. Two-sided Fisher’s exact tests were used for overrepresentation analyses. For qPCR, western blot, and fluorescent-based analyses, results are based on the combination of data from three separate transductions. Two-tailed Student’s t-tests were used to analyze results from these experiments, except for quantification of CYFIP1 mRNA and protein levels, where one-tailed Student’s t-tests were used. Fold enrichment was calculated as the fraction of DEGs that are within a given category (ex. FMRP targets) divided by the fraction of total expressed genes that are within that same given category. All results presented in graphs are expressed as fold change ± SEM. The number of samples used in each experiment is included in the figure legends.

## Supporting Information

S1 Table Quality control metrics for RNA-seq data. (XLSX)

S2 Table Complete list of expressed genes in at least one donor line (TPM>1 in non-silencing control samples) from RNA-seq experiments. (XLSX)

S3 Table Complete list of genes identified as differentially expressed in response to *CYFIP1* knockdown with corresponding status with regard to FMRP target, postsynaptic density, schizophrenia, and epilepsy. (XLSX)

S4 Table Lists of FMRP target and PSD genes along with disease and trait-related genes used for enrichment analyses. (XLSX)

S5 Table Postsynaptic density genes dysregulated in response to *CYFIP1* knockdown are involved in cytoskeletal remodeling. (XLSX)

S6 Table Results for seven diseases or traits not known to be associated with BP1-2 deletion status. (XLSX)

S7 Table Results from gene ontology analyses for schizophrenia genes dysregulated in response to *CYFIP1* knockdown. (XLSX)

S8 Table All significant ToppGene results for genes dysregulated in response to *CYFIP1* knockdown. (XLSX)

S9 Table Clinical information for BP1-2 deletion and BP1-2 neutral subjects from which lymphoblastoid cell lines were generated. (XLSX)

S10 Table List of primers used for qPCR. (XLSX)
